# Comparison of Direct Oral Anticoagulants and Warfarin in the Prevention of Stroke in Patients With Valvular Heart Disease: A Meta-Analysis

**DOI:** 10.7759/cureus.28763

**Published:** 2022-09-04

**Authors:** Saima Batool, Sandipkumar S Chaudhari, Tanveer Ahamad Shaik, Sandesh Dhakal, Zubair Ahmad Ganaie, Muhammad Abu Zar Ghaffari, Faraz Saleem, Areeba Khan

**Affiliations:** 1 Internal Medicine, Hameed Latif Hospital , Lahore, PAK; 2 Medicine, Lions General Hospital, Mehsana, IND; 3 Medicine, General Hospital, Vadnagar, IND; 4 Cardiovascular Medicine, University of Louisville School of Medicine, Louisville, USA; 5 Medicine, College of Medical Sciences, Bharatpur, NPL; 6 Internal Medicine, Holy Family Red Crescent Medical College, Srinagar, IND; 7 Internal Medicine, Akhtar Saeed Medical and Dental College, Lahore, PAK; 8 Critical Care Medicine, United Medical and Dental College, Karachi, PAK

**Keywords:** meta-analysis, stroke, direct oral anticoagulants, valvular heart disease, warfarin

## Abstract

Warfarin is the standard of care, and direct oral anticoagulants (DOACs) are a group of newer drugs to prevent stroke in patients with valvular heart disease. The aim of this meta-analysis is to compare the efficacy and safety of DOACs and warfarin in the prevention of stroke in patients with valvular heart disease (VHD). The current meta-analysis was conducted using the standards developed by the Preferred Reporting Items for Systematic Reviews and Meta-Analyses (PRISMA) recommendation. The databases from the Cochrane library, PubMed, and Excerpta Medica database (EMBASE) were used to search for relevant articles without placing restrictions on the year of publication. Outcomes assessed in the current meta-analysis included a number of patients with stroke or systemic embolism, patients having myocardial infarction during the study period, patients with major bleeding events, and patients who died due to any reason. Overall, five studies were included in the current meta-analysis. Direct oral anticoagulants were associated with a lower risk of stroke or systemic embolism in patients with VHD (relative risk (RR): 0.75, 95% confidence interval (C)I: 0.60 to 0.94). The risk of major bleeding events is 31% lower in patients receiving DOAC compared to patients receiving warfarin (RR: 0.69, 95% CI: 0.58 to 0.83). No significant difference was found between the two groups in terms of all-cause mortality and myocardial infarction. The current meta-analysis shows that DOACs were associated with a lower risk of stroke or systemic embolism as compared to warfarin in patients with VHD. Besides this, the risk of major bleeding events was also lower in patients receiving DOACs compared to patients receiving warfarin. No significant differences were reported in terms of myocardial infarction and all-cause mortality between the two groups.

## Introduction and background

Valvular heart disease (VHD) can enhance the risk of stroke and systemic embolic events (SSEE) and atrial fibrillation (AF) [1]. Thus, anticoagulants are usually administered to patients with VHD. Warfarin was the standard of care and it was the only oral medication available before the development of novel oral anticoagulants (NOACs) [2]. It can inhibit the formation of coagulation factors related to vitamin K, such as factors II, VII, IX, and X, and can help avoid thromboembolism. Novel oral anticoagulants are newer drugs for the prevention and treatment of thromboembolism. There are two major classes of NOACs, namely direct thrombin inhibitors (dabigatran) and factor Xa inhibitors (rivaroxaban, edoxaban, and apixaban) [3]. When compared to warfarin, NOACs are more effective, need low monitoring, and have fewer adverse effects. However, they lack an antidote, are only occasionally used in individuals with renal impairment, and are more expensive [4]. Even though NOAC has a better safety profile but its efficacy in valvular AF remains unclear [5]. Thus, in patients with a mechanical prosthetic heart valve, warfarin is the only recommended anti-coagulant for the prevention of SSEE [6].

It is conjectured that the formation of thrombus in patients with non-valvular AF may be different than that of patients with VHD. Thrombi commonly form in the left atrial appendage in AF patients [7]. Due to non-physiologic blood flow patterns caused by the prosthesis, thrombi frequently develop on the prosthesis or in the left atrium in patients with mechanical prosthetic heart valves [7]. The risk of thrombosis in patients with bioprosthetic heart valves is lower, but not zero. In patients who receive a bioprosthetic valve, the risk of thrombosis increases in the setting of mitral stenosis or concurrent AF [8].

The United States Food and Drug Administration (FDA) and current European Medicines Agency (EMA) approval of rivaroxaban, edoxaban, dabigatran, and apixaban only include the indications of non-valvular AF patients [9]. However, data is there to support the use of these medications in patients with certain types of valvular AF as well. Patients with mild mitral regurgitation, aortic regurgitation, tricuspid regurgitation, aortic stenosis, and prior valve surgery were included in sub-analyses of the direct oral anti-coagulant (DOAC) trials for AF [9].

As the prevalence of valvular heart disease rises in the general population and with age, large numbers of patients are likely to have underlying native VHD along with AF [10]. The choice of anticoagulant to utilize in these patients may be influenced by the simultaneous occurrence of AF and native valvular heart disease. The aim of this meta-analysis is to compare the efficacy and safety of DOACs and warfarin in the prevention of stroke in patients with valvular heart disease.

## Review

Methodology

The current meta-analysis was conducted using the standards developed by the Preferred Reporting Items for Systematic Reviews and Meta-Analyses (PRISMA) recommendations.

Search Strategy and Study Selection

The databases of the Cochrane library, PubMed, and Excerpta Medica database (EMBASE) were used to search for relevant articles without placing restrictions on the year of publication. Pre-defined search terms were used to identify relevant randomized control trials including “novel oral anticoagulants”, “warfarin”, “valvular heart disease”, “stroke prevention”, and “efficacy and safety”. To be eligible for inclusion in the current meta-analysis, studies had to fulfill the following pre-defined inclusion criteria: randomized clinical trials (RCTs) that compared DOAC and warfarin in individuals aged 18 years or more and with valvular heart disease. Articles compared DOAC and warfarin in patients without valvular heart disease were excluded. In addition, studies with a follow-up period of less than one month were also not included in the current meta-analysis. Lastly, observational studies, reviews, case reports, and non-randomized clinical trials were also excluded from the current meta-analysis.

Two authors independently participated and completed the process of initial search followed by a title and abstract screening. The full text of all eligible articles was retrieved and assessed for eligibility criteria. Disagreements were resolved between two reviewers through discussion or involvement of a third author.

Data Extraction

Data were extracted from relevant articles using a structured form developed in Microsoft Excel (Microsoft Corporation, Redmond, WA, USA). Two authors extracted the data independently. Any disagreement was resolved between the two authors through discussion or involvement of the third author. Study characteristics that were excluded from the articles included the name of the first author, year of publication, intervention, sample size, follow-up period, and characteristics of participants (mean age and gender).

Outcomes

Outcomes assessed in the current meta-analysis included a number of patients with stroke or systemic embolism, a number of patients having myocardial infarction during the study period, a number of patients with major bleeding events, and a number of patients who died due to any reason (all-cause mortality).

Assessment of Risk of Bias

The risk of bias was evaluated by two authors separately for each publication. Any discrepancy between the two authors was settled by consensus or, if necessary, discussion with a third investigator. The Cochrane Risk of Bias tool was used to evaluate the risk of bias for each randomized control trial. Six domains were assessed to evaluate the risk of bias including random sequence generation, allocation concealment, blinding of personnel and participants, blinding of outcomes assessment, incomplete outcome data, selective outcome reporting, and other biases. Each potential bias factor was rated as low, unclear, or high.

Statistical Analysis

Statistical analysis was done using the Cochrane collaboration review manager software, Revman version 5.4.0 (Cochrane, London, UK). Random-effects or fixed-effects meta-analysis model was used and forest plots were drawn to present the pooled estimates of the risk ratio (RR) and 95% confidence interval (CI). A p-value of less than 0.05 was considered statistically significant. Heterogeneity between the study results was assessed using I-square statistics. The random effect model was used if I2 was more than 50%, while the fixed effect model was used if I2 was less than or equal to 50%. For statistical testing of heterogeneity, the Cochrane-Q test was used. A p-value ≤ 0.1 was considered statistically significant for heterogeneity. Egger's test was used to assess the publication bias. A P-value<0.05 was considered significant for publication bias.

Results

**Figure 1:**
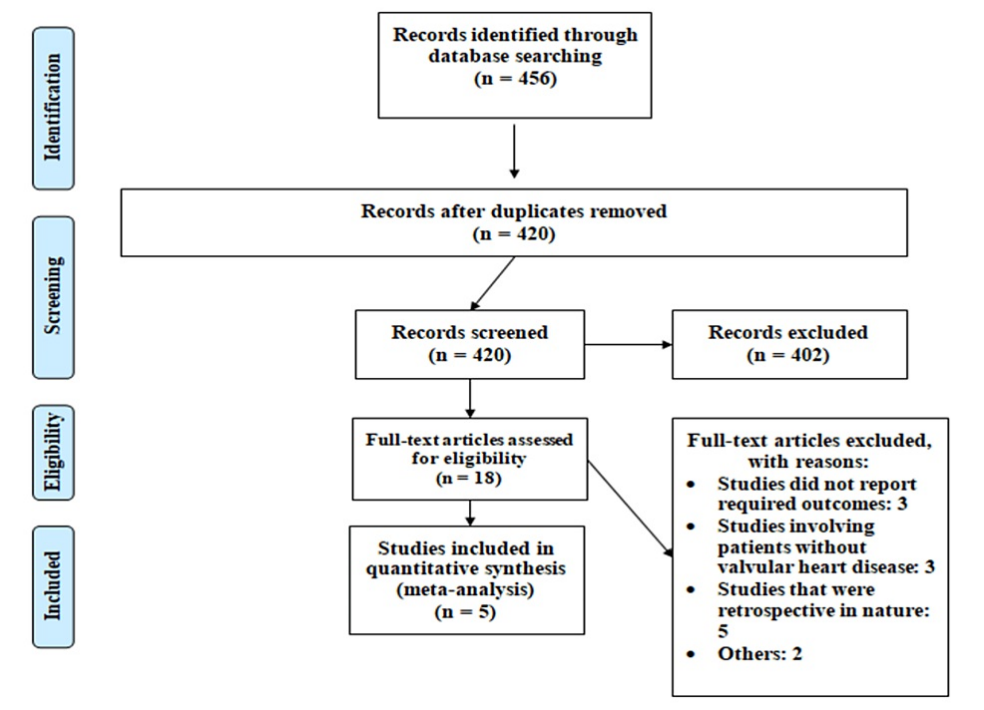
PRISMA flowchart of the selection of studies PRISMA: Preferred Reporting Items for Systematic Reviews and Meta-Analyses

Figure 1 shows the PRISMA flowchart for the selection of studies. Overall, 456 articles were retrieved from online databases searching. After removing duplicates, title and abstract screening of 420 articles were done. We were able to exclude 402 articles based on the title and abstract of those articles. Full-text screening of all remaining articles was performed, which excluded 13 more articles as per the eligibility criteria. Then, data from five eligible articles were extracted.

Table 1 shows the characteristics of the included studies. Out of all included studies, four were multicenter [11-14], while one was conducted in a single center only [15]. The follow-up period of all included studies ranged from three Months to 33.6 Months. 

**Table 1:** Characteristics of the included studies

First Author	Year	Setting	Groups	Sample size	Follow-up	Mean age in years	Male n (%)
Avezum et al. [11]	2014	Multicenter	Apixaban	2438	21.8 Months	71	2872 (59.7)
			Warfarin	2370			
Caterina et al. [12]	2017	Multicenter	Edoxaban	1869	33.6 Months	71.8	1631 (57.8)
			Warfarin	955			
Eikelboom et al. [13]	2013	Multicenter	Dabigartan	168	3 Months	55.8	163 (64.7)
			Warfarin	84			
Guimaraes et al. [14]	2020	Multicenter	Rivaroxaban	500	12 Months	59.3	398 (39.6)
			Warfarin	505			
Duraes et al. [15]	2016	Single center	Dabigartan	15	3 Months	47.3	10 (37.0)
			Warfarin	12			

**Figure 2:**
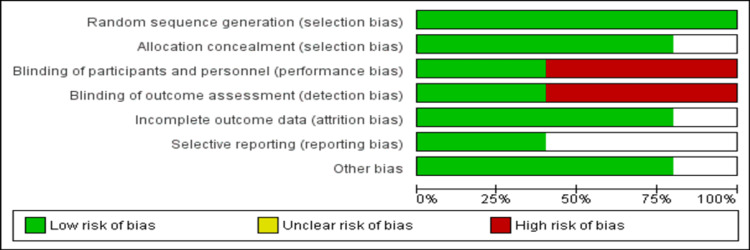
Risk of bias assessment

Figure 2 shows the risk of bias assessment of all included studies. The overall risk of bias is moderate. In two articles, there was unblinding of participants as well as investigators, while three studies were open-label.

Comparison of DOACs and Warfarin

**Figure 3:**
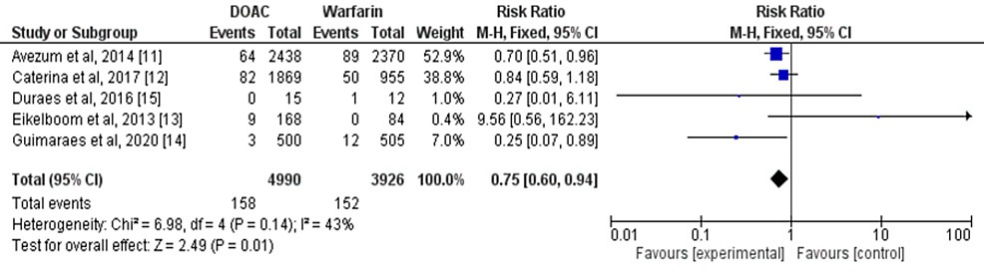
Forest plot of comparison of DOACs and warfarin on the risk of stroke in patients with VHD Sources: References [11-15] DOACs: Direct oral anticoagulants, VHD: Valvular heart disease, CI: Confidence interval

Overall, five studies compared the incidence of stroke or systemic embolism between patients who received DOAC and warfarin [11-15]. The DOACs were associated with a lower risk of stroke or systemic embolism in patients with VHD (RR: 0.75, 95% CI: 0.60 to 0.94) as shown in Figure 3. Heterogeneity among the study results was low (I2=43%). Cochran Q test found no significant heterogeneity among the study results (p=0.14).

**Figure 4:**
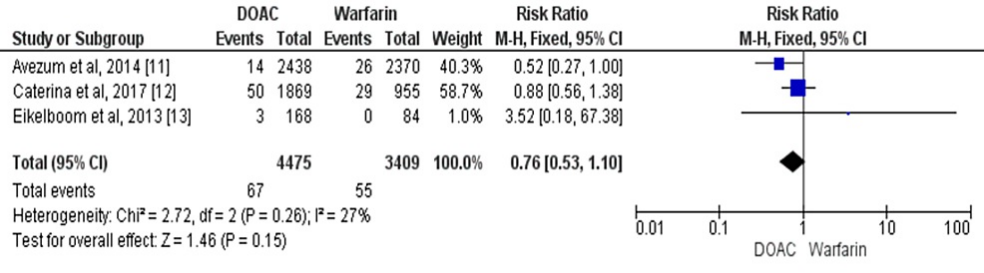
Forest plot of comparison of DOACs and warfarin on the risk of myocardial infarction in patients with VHD Sources: References [11-13] DOACs: Direct oral anticoagulants, VHD: Valvular heart disease, CI: Confidence interval

Overall, three studies compared the risk of myocardial infarction between DOACs and warfarin [11-13]. No significant difference was there in the risk of myocardial infarction between the two groups (RR: 0.76, 95% CI: 0.53 to 1.10) as shown in Figure 4. No significant heterogeneity was found among the study results (p=0.26).

**Figure 5:**
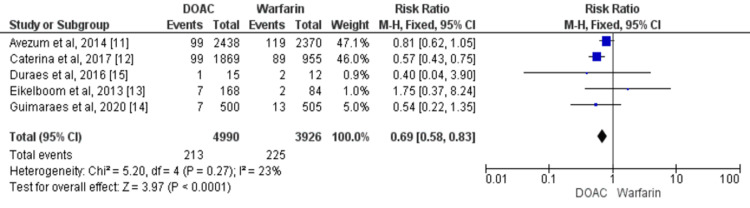
Forest plot of comparison of DOACs and warfarin on the risk of major bleeding events in patients with VHD Sources: References [11-15] DOACs: Direct oral anticoagulants, VHD: Valvular heart disease, CI: Confidence interval

Overall, five studies involving 8916 patients with VHD compared the incidence of major bleeding events between DOACs and warfarin [11-15]. The risk of major bleeding events is 31% lower in patients receiving DOAC compared to patients receiving warfarin (RR: 0.69, 95% CI: 0.58 to 0.83) as shown in Figure 5. Heterogeneity among the study results was low (I2=23%). No significant heterogeneity was there among the study results as the p-value was more than 0.1.

**Figure 6:**
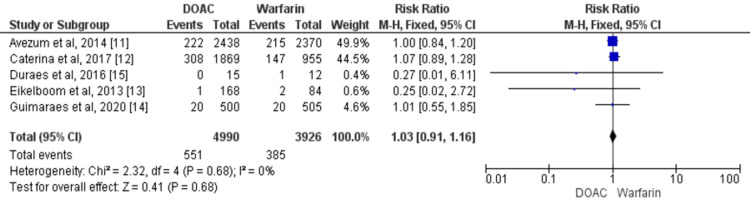
Forest plot of comparison of DOACs and warfarin on the risk of all-cause mortality among patients with VHD Sources: References [11-15] DOACs: Direct oral anticoagulants, VHD: Valvular heart disease, CI: Confidence interval

Overall, five studies involving 8916 patients with VHD compared all-cause mortality between the two groups [11-15]. No significant difference was found in terms of all-cause mortality between patients who received DOACs and patients who received warfarin (RR: 1.03, 95% CI: 0.91 to 1.16) as shown in Figure 6. No significant heterogeneity was found among the study results (p=0.68).

Publication Bias

The Egger’s test showed that no indication of small study effects was there (p=0.233) for the pooled effect estimates of the primary efficacy outcome i.e., stroke or systemic embolism.

Discussion

This meta-analysis shows that DOACs were associated with a lower risk of stroke or systemic embolism compared to patients receiving warfarin. The risk of major bleeding events was also lower in patients receiving DOACs compared to warfarin. However, no significant difference between the two groups was there in terms of all-cause mortality and myocardial infarction.

Warfarin has been long known to decrease the risk of stroke in patients with AF. However, frequent dose adjustment and monitoring are required and might be inconvenient for patients [16]. The use of DOACs has been approved for the prevention of stroke in patients with AF [17]. In daily practice, DOACs are preferred over warfarin because of the lack of monitoring required and the better safety profile [16]. However, the scarcity of evidence related to the use of DOACs in patients with VHD is one of the major issues because of a small number of studies. For example, the safety and efficacy of DOACs seem to be different in patients with aortic stenosis as compared to other VHDs like aortic regurgitation or mitral regurgitation [18].

One of the significant barriers in determining if DOACs are superior to warfarin in patients with valvular atrial fibrillation is the definition of non-valvular AF which is inconsistent and not universally defined [19]. Since there is no accepted definition of non-valvular AF, there has been an ongoing discussion about which patients with AF and underlying VHD should receive a NOAC. In the historic NOAC studies, patients with VHD had mitral regurgitation as the most frequent valvular lesion [19].

Post-hoc analysis of the patients with VHD and AF in the trial conducted by Avezum et al. [11] showed that apixaban was superior to warfarin in the prevention of stroke. This is in line with the overall study findings that showed apixaban to be superior to warfarin. The best-known data that supports the use of NOAC in patients with AF and valvular heart disease is found in this post hoc subgroup analysis of the seminal NOAC trials [11]. Noseworthy et al. conducted a retrospective study that reported similar findings being NOACs superior to warfarin [20]. This study also reported fewer events of major bleeding in patients receiving NOAC. The American College of Cardiology valvular heart disease guidelines recommended that NOACs be utilized in preference over warfarin in VHD and AF patients (particularly mitral regurgitation, tricuspid valve disease, and aortic valve disease) based on the studies included in the current meta-analysis [8]. Due to the small number of mitral stenosis patients that were included in the seminal NOAC research [11], the data supporting the use of NOACs in mitral stenosis patients remains uncertain. Additionally, patients with prosthetic valves were generally excluded from the VHD patient subgroup analysis in the seminal NOAC investigations by Avezum et al. [11]. Therefore, due to a paucity of data, the use of NOACs in individuals with atrial fibrillation, mitral stenosis, or prosthetic valves should be discouraged.

With the findings of the current meta-analysis, DOACs were shown to be more effective in preventing stroke and systemic embolism in patients with VHD. To date, warfarin is the main anti-coagulant for valve surgery and VHD [21]. For the mechanical valve, warfarin was the sole option available, and the bioprosthetic valve requires adjuvant anti-platelet therapy [21]. Many ongoing studies are assessing the efficacy of NOAC in VHD. Due to limited evidence, NOAC can be used in some patients with VHD as long as it is not contraindicated. It is ultimately the decision of physicians to prescribe NOAC to patients by balancing the risk and benefits. Physicians should choose the medicine based on an individualized analysis of the valvular pathology and functional status of the patient and provide a management plan that is appropriate for the patient.

One of the major limitations of this current meta-analysis is that all the included studies are based on post hoc analysis of randomized control trials. In most of the included studies, the numbers of patients with VHD were quite small and were not provided with enough power for detecting the true benefits of DOACs over warfarin. Additionally, patient co-morbidities and underlying valvular diseases varied in the aforementioned research, making it difficult to generalize the findings. Besides, the choice of materials for a valve for patients undergoing valve surgery with anticoagulation may have a different impact on the prognosis. Valvular heart disease is a wider category of several heart conditions that may have distinct treatments, pathogenesis, etiologies, and prognosis. There is insufficient literature to emphasize the sub-group analysis. For analyzing this particular aspect, more prospective studies are required to focus on valvular heart disease to determine whether DOACs are beneficial over warfarin in these patients.

## Conclusions

The current meta-analysis shows that DOACs are associated with a lower risk of stroke or systemic embolism as compared to warfarin in patients with VHD. Also, the risk of major bleeding events was lower in patients receiving DOACs compared to patients receiving warfarin. No significant differences were reported in terms of myocardial infarction and all-cause mortality between the two groups. More prospective future studies need to be conducted to assess the efficacy and safety of DOACs in patients with VHD to reduce the risk of major events in these patients.
